# Common Cold Symptoms in Children: Results of an Internet-Based Surveillance Program

**DOI:** 10.2196/jmir.2868

**Published:** 2014-06-19

**Authors:** Emanuel Troullos, Lisa Baird, Shyamalie Jayawardena

**Affiliations:** ^1^Pfizer Consumer HealthcareGlobal Clinical DevelopmentMadison, NJUnited States

**Keywords:** common cold, pediatric, sleep, surveillance, symptoms

## Abstract

**Background:**

Conducting and analyzing clinical studies of cough and cold medications is challenging due to the rapid onset and short duration of the symptoms. The use of Internet-based surveillance tools is a new approach in clinical studies that is gradually becoming popular and may become a useful method of recruitment. As part of an initiative to assess the safety and efficacy of cough and cold ingredients in children 6-11 years of age, a surveillance program was proposed as a means to identify and recruit pediatric subjects for clinical studies.

**Objective:**

The objective of the study was to develop an Internet-based surveillance system and to assess the feasibility of using such a system to recruit children for common cold clinical studies, record the natural history of their cold symptoms, and determine the willingness of parents to have their children participate in clinical studies.

**Methods:**

Healthy potential subjects were recruited via parental contact online. During the 6-week surveillance period, parents completed daily surveys to record details of any cold symptoms in their children. If a child developed a cold, symptoms were followed via survey for 10 days. Additional questions evaluated the willingness of parents to have their children participate in a clinical study shortly after onset of symptoms.

**Results:**

The enrollment target of 248 children was reached in approximately 1 week. Children from 4 distinct geographic regions of the United States were recruited. Parents reported cold symptoms in 163 children, and 134 went on to develop colds. The most prevalent symptoms were runny nose, stuffed-up nose, and sneezing. The most severe symptoms were runny nose, stuffed-up nose, and sore/scratchy throat. The severity of most symptoms peaked 1–2 days after onset. Up to 54% of parents expressed willingness to bring a sick child to a clinical center shortly after the onset of symptoms. Parents found the Internet-based surveys easy to complete.

**Conclusions:**

Internet-based surveillance and recruitment can be useful tools to follow colds in children and enroll subjects in clinical studies. However, study designs should account for a potentially high dropout rate and low rate of adherence to study procedures.

## Introduction

The common cold is one of the most prevalent illnesses in the world [1]. Adults may experience 2-5 colds per year, and children may experience 7-10 colds per year [2]. Owing to the high rate of incidence, especially among children, the common cold creates a significant economic and social burden [1,3,4]. Symptoms of the common cold in children typically reach peak intensity shortly after the onset of illness [5]. Symptom duration is approximately 7-10 days but may range from 2-14 days [3,4]. Diagnosis of the common cold can be problematic in young children and infants who are not able to communicate their symptoms. Because colds are of limited duration and clinical studies of cough and cold medications rely on self-reported assessment of symptoms, the conduct and analysis of such studies is highly challenging. Evaluating the efficacy of over-the-counter cough and cold products is therefore equally challenging. Given these hurdles, optimizing clinical design is paramount to fully assess the efficacy of current and future cough and cold products. One design element that has proven important in cold studies is the ability to enroll subjects at the earliest stages of a cold [3,5,6]. As part of an initiative to assess the safety and efficacy of cough and cold ingredients in children 6-11 years of age, a surveillance program was proposed as a means to identify and recruit pediatric subjects for participation in clinical studies. Surveillance systems have been used to monitor daily health and to study infectious disease dynamics in a daycare setting [7,8]. Engaging parents with a surveillance program was thought to be a way of generating a pool of potential patients for recruitment into a clinical study within the first 2 days of the appearance of cold symptoms. The present study was undertaken to develop and test Internet-based surveillance methodology designed to recruit and prescreen children aged 6-11 years, monitor these children on a daily basis for the first onset of cold symptoms, determine the feasibility of enrolling them into a clinical study within approximately 24 hours of the onset of cold symptoms, identify potential recruitment issues, and assess the proposed recruitment strategy.

## Methods

### Subjects

Eligible subjects were children 6-11 years of age. We required a history of 3 or more colds per winter season in either of the past 2 winters as reported by parents (“parent” includes parents and legally authorized representative), and/or a history of living with 3 or more siblings or other children, in order to increase the likelihood that the child would develop a cold during the 6-week surveillance period. Each subject’s parent was required to have access to a home computer with an Internet connection. Major exclusion criteria included cardiovascular or thyroid disease, asthma requiring daily medication, chronic bronchitis, glaucoma, use of sedatives or tranquilizers, use of monoamine oxidase inhibitors, smoking, and history of attention deficit disorder or attention deficit hyperactivity disorder. Concomitant medications, apart from these exclusions, were allowed. Only 1 child per household was permitted into the study. The study was conducted in accordance with the requirements specified in the US Code of Federal Regulations (21 CFR Parts 50, 56, 312) and the International Conference on Harmonization Good Clinical Practice Guidelines, and informed consent was provided by parents before initiation of any study procedures. The final protocol and informed consent documentation were reviewed and approved by an independent institutional review board (Copernicus Group).

### Study Design

This was a noninterventional surveillance study in school-age children (ages 6-11 years). Potential subjects were identified through the resources of a patient recruitment provider with an extensive database of individuals who had chosen to receive new health information and clinical study invitations. Letters were sent to individuals in the network in 4 geographically diverse metropolitan areas (Austin, TX; Boston, MA; Portland, OR; Raleigh, NC) informing them of the study and directing them to a website if they wished to obtain additional information about enrolling their child in the study. Screening and evaluations were performed online by parents. Once the subjects were enrolled, parents recorded the presence or absence of 10 cold symptoms each evening for up to 6 weeks by completing a Well Child Daily Survey (WCDS) (see Multimedia Appendix 1). Cold symptoms included in the survey were runny nose, stuffed-up nose, sneezing, sore/scratchy throat, dry cough (no mucus), wet cough (with mucus), chest congestion, headache, muscle/body ache, and feverishness/chilliness. On the first day that parents reported their child was experiencing cold symptoms, they were asked questions designed to evaluate their willingness and availability to bring their child to a clinic and to participate in a clinical study of cough/cold medication. If the child developed a cold, the parent recorded details of the symptoms by using a Daily Cold Symptom Severity Survey (DCSSS; see Multimedia Appendix 2). Parents rated each of the 10 individual cold symptoms on a 4-point categorical rating scale. Additionally, parents recorded whether they believed the symptoms were related to a cold as opposed to a different upper respiratory illness, such as allergy or flu. At the end of the surveillance period, parents were asked to complete an End of Study Survey (EOSS) that included questions about the effect of cold symptoms on the child’s sleep and the ability of the parent to use the Internet-based tools to identify and evaluate cold symptoms and comply with the requirements of the study. Because no therapeutic intervention was planned, safety and adverse event data were not collected (Multimedia Appendix 1 and 2).

### Statistical Analysis

No formal hypotheses were tested; all outcome measures are described by summary statistics.

## Results

### Subject Characteristics

Between January 5 and January 11, 2011, 2543 parents entered the system by visiting the website. Of those, 425 completed the prescreening questionnaire, 346 completed the informed consent form, and 248 children were enrolled. Demographic characteristics of the study population are presented in Table 1.

**Table 1:** Demographic characteristics of enrolled children (N=248).

Characteristic		n (%)
**Gender**		
	Male	160 (64.5)
	Female	88 (35.5)
**Age, years**		
	6	36 (14.5)
	7	60 (24.2)
	8	61 (24.6)
	9	48 (19.4)
	10	23 (9.3)
	11	20 (8.1)
**Race**		
	White	183 (73.8)
	Black	34 (13.7)
	American Indian or Alaskan native	6 (2.4)
	Asian	5 (2.0)
	Mixed race	16 (6.5)
	Did not answer	4 (1.6)
**Ethnicity**		
	Not Hispanic or Latino	222 (89.5)
	Hispanic or Latino	23 (9.3)
	Did not answer	3 (1.2)
**Geographic region**		
	Austin, TX	56 (22.6)
	Boston, MA	71 (28.6)
	Portland, OR	67 (27.0)
	Raleigh, NC	54 (21.8)
**Number of other children in the home**		
	0	72 (29.0)
	1	31 (12.5)
	2	43 (17.3)
	≥3	102 (41.1)
**Highest education level of parent**		
	Did not graduate high school	4 (1.6)
	High school graduate (or equivalent)	35 (14.1)
	Some college education	85 (34.3)
	Bachelor’s degree	92 (37.1)
	Master’s degree	26 (10.5)
	Doctorate degree	5 (2.0)
	Did not answer	1 (0.4)
**Household income**		
	<$25,000	18 (7.3)
	$25,001–$50,000	53 (21.4)
	$50,001–$100,000	138 (55.6)
	>$100,000	37 (14.9)
	Did not answer	2 (0.8)

### Survey Results

#### Natural History of Colds in Enrolled Children

**Figure 1:**
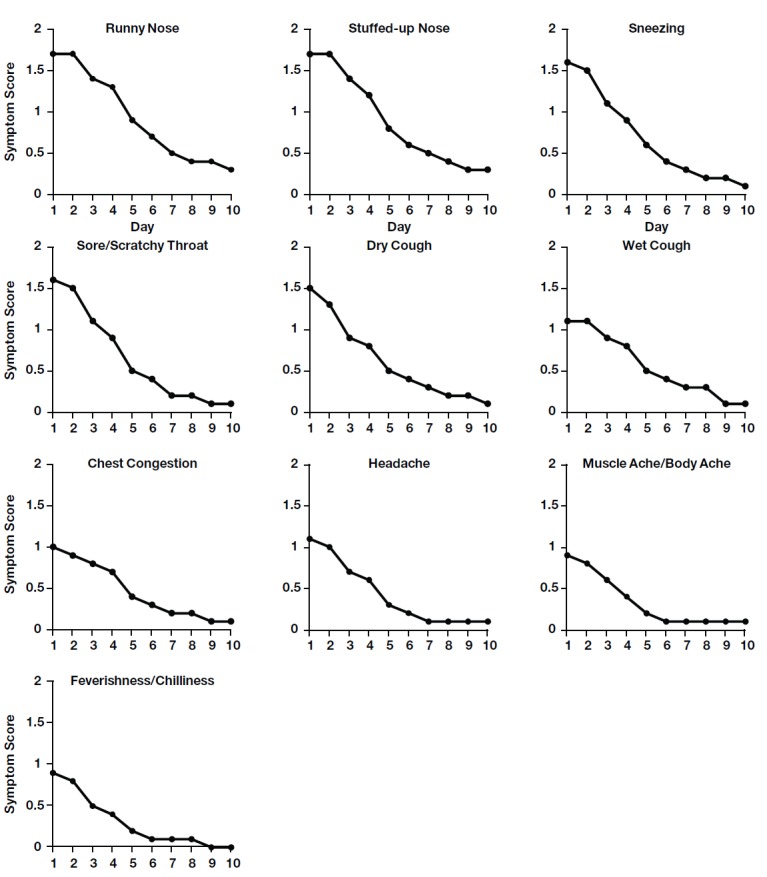
Average daily severity of individual cold symptoms. Parents of children with colds (n=134) were asked to score symptoms on a scale from 0 (not present) to 3 (severe). To calculate the average daily severity, a value of zero was assigned for those subjects who did not provide a symptom severity rating on a given day during the 10-day follow-up period because the parent was not required to complete the symptom severity questionnaire after all symptoms had resolved.

Using the WCDS, parents reported cold symptoms in 65.7% (163/248) of the enrolled children. Using the DCSSS, parents reported that 54.0% (134/248) of the children developed colds and asserted in their opinion that the symptoms were not related to a different upper respiratory condition, such as allergy or flu. In this study, development of a cold was defined as at least 1 entry in the DCSSS with symptoms present and confirmation that the parent believed the symptoms were related to a cold. Symptoms were ranked in severity on a 4-step scale: “symptom not present”, “mild”, “moderate”, and “severe.” Of the 10 symptoms evaluated, the most commonly reported as severe were runny nose (65/134, 48.5%), stuffed-up nose (65/134, 48.5%), and sore/scratchy throat (62/134, 46.3%). All symptoms were most severe in the first 1-2 days after onset and declined thereafter (Figure 1). Moderate-to-severe runny nose in the first 24 hours after onset was reported in 64.2% (86/134) of the children who suffered colds; moderate-to-severe chest congestion within 48 hours was reported in 51.5% (69/134) of the children.

**Figure 2:**
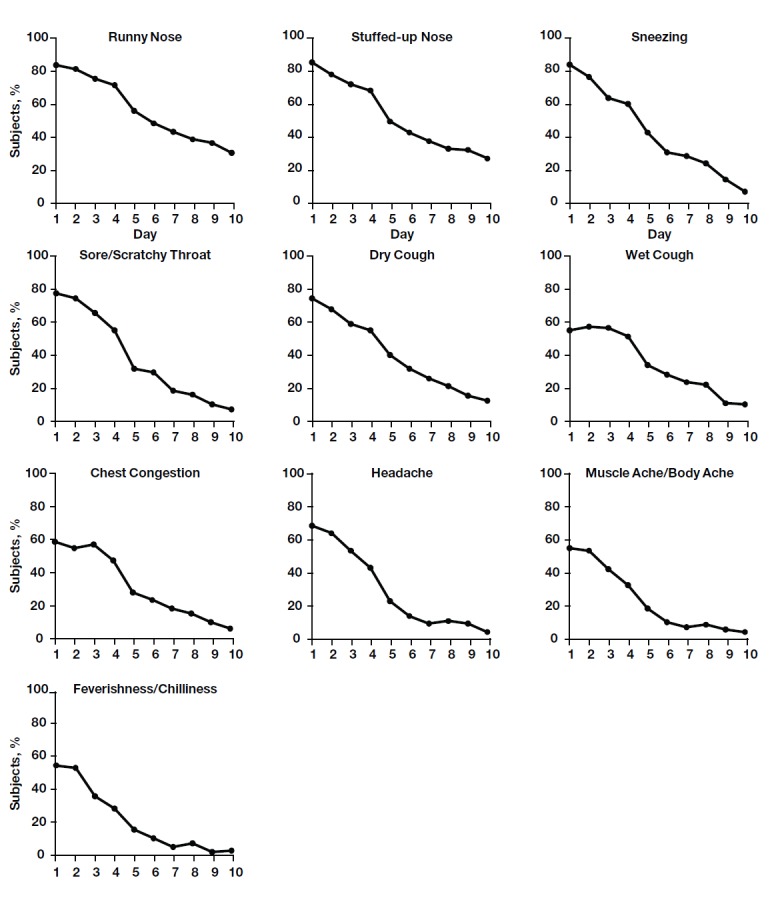
Average daily prevalence of individual cold symptoms. From the day of onset, parents of children with colds (n=134) were asked to report whether a symptom was present or absent.

The most prevalent symptoms among children with colds, regardless of severity, were runny nose, stuffed-up nose, dry cough, sore throat/scratchy throat, and sneezing; at least 75% of subjects experienced these symptoms on Day 1. The time course of symptom prevalence had a profile similar to that of symptom severity, with a peak on Day 1 or 2 followed by a decline (Figure 2). The least prevalent symptoms were muscle ache/body ache and feverishness/chilliness, which were reported in 55.2% (74/134) and 54.4% (73/134) of children, respectively, on Day 1. Most symptoms were largely resolved by Day 10 (reported in <15% of children).

#### Effect on Sleep

Of those parents whose children developed colds during the surveillance period, 81.3% (109/134) completed the EOSS. An additional aspect of the survey was to assess the effect of a cold on a child’s sleep, and the EOSS asked parents to record the effect of a cold on their child’s sleep on the night that cold symptoms were at their worst. Of the parents who completed the sleep questionnaire for a child who developed a cold, nearly two-thirds (55/93, 59.1%) reported that their child’s sleep quality was somewhat worse than usual. Nearly three-quarters of parents (69/93, 74.2%) reported that their child had at least some difficulty falling asleep. A large majority of parents (81/93, 87.1%) reported that their child woke at least once during the night.

Parents reported that the majority of children who developed colds were given medication for their cold symptoms. A similar percentage of children were given nonprescription medicine (50/134, 37.3%) or prescription medicine (49/134, 36.6%). In addition, 12.7% (17/134) were given both non-prescription and prescription medication.

### Parental Willingness to Participate in Clinical Studies

When a parent observed that their child was experiencing the first occurrence of symptoms and they believed that the child was experiencing a cold, the parent was asked a series of questions regarding their willingness to comply with certain procedures that might be necessary for participation in a clinical study; 65.7% (163/248) parents replied (Table 2). Among parents who responded on the same day that symptoms appeared, 26.4% (43/163) expressed willingness and availability to bring their child to a clinical site. Including respondents who answered within 1 day of the appearance of symptoms, the number of parents who would be willing/available to bring the child to a clinical site rose to 54.0% (88/163). Only 12.3% (20/163) of the respondents indicated a willingness to allow the child to remain home from school. Somewhat more parents, 29.4% (48/163) reported willingness to score symptoms and administer a study medication to the child during lunch breaks at school, and 30.7% (50/163) reported that they were willing to allow the child to remain home from school and/or score symptoms and administer study medications at school.

**Table 2:** Parent willingness to participate in clinical study procedures (N=163).

Procedure		Willing, n (%)	Not willing, n (%)
**Visit a clinical site (response on same day as the appearance of symptoms in the child)**			
	Total	43 (26.4)	120 (73.6)
	Before 11 am the next day	14 (8.6)	
	Before the clinical site closes the next day	15 (9.2)	
	Within the next 3 days	21 (12.9)	
	Within the next 5 days	20 (12.3)	
**Visit a clinical site (response within 1 day of appearance of symptoms in the child)**			
	Total	88 (54.0)	75 (46.0)
	Before 11 am the next day	29 (17.8)	
	Before the clinical site closes the next day	35 (21.5)	
	Within the next 3 days	32 (19.6)	
	Within the next 5 days	30 (18.4)	
Permit a child to remain out of school to participate in a study		20 (12.3)	143 (87.7)
Score symptoms and administer medication to the child at school		48 (29.4)	115 (70.6)
Permit a child to remain out of school and/or score symptoms and administer medication to the child at school		50 (30.7)	113 (69.3)

### Ease of Participation

In addition to evaluating the quality of sleep in enrolled children, the EOSS asked parents to rate how easy or difficult it was for them to participate in this Internet-based program. Among parents who completed the EOSS, 89.0% (97/109) reported that it was easy or very easy to use the website on a daily basis, and the majority were able to complete all surveys with little difficulty. Respondents consistently reported that it was easy or very easy to rate individual cold symptoms in their children (78/109, 71.6% to 93/109, 85.3%, depending on the symptom), and 85.3% (93/109) felt that it was easy or very easy to decide whether the child had a cold rather than some other illness.

## Discussion

### Principal Findings

This study examined the suitability of an Internet-based surveillance program to recruit and prescreen school-age children for common cold clinical studies, monitor the natural history of their colds, and evaluate their parents’ willingness to quickly enroll them in clinical studies. The results demonstrated that the Internet-based methodology was efficient and effective. Of enrolled children, 54.0% (134/248) developed colds, and parents monitored the severity and duration of 10 individual symptoms. The single most prevalent symptom was runny nose, which was reported in 94.8% (127/134) of children with colds. Other highly prevalent symptoms were stuffed-up nose, sneezing, dry cough, and scratchy/sore throat, which were reported in 86.6% (116/134) or more of children. The symptoms that were most highly prevalent also were frequently rated as severe. Runny nose and stuffed-up nose remained present after 6 days or more in 85.5% (106/124) or more of children who experienced those symptoms. The symptoms that were least prevalent and resolved most quickly were muscle/body ache and feverishness/chilliness. Because these symptoms are more commonly associated with influenza, this observation suggests that children were suffering colds and that their parents were able to differentiate between the 2 conditions.

The patterns of symptom prevalence, severity, and duration observed in the present study are in accord with those previously reported in adults and children [2,4,9-11]. In a study of colds in children, runny nose and nasal congestion were found to be the most common symptoms and 73% of children remained symptomatic 10 days after onset of the cold [5]. An early study of colds in adults found that runny nose, sneezing, and sore throat were the most common symptoms and the mean duration of a cold was 7.4 days [11]. A more recent study reported that runny nose, stuffy nose, and sore throat were the most bothersome symptoms in adults; median duration of symptoms was 11 days [9]. The severity of cold symptoms has been reported to reach a peak 2-3 days after onset [2,10].The results presented here are consistent with these earlier investigations and suggest that the natural history of colds is similar in adults and in children and that an Internet-based program is an effective way to collect data on cold symptoms.

The enrollment data from the present study indicate that the Internet-based strategy was effective at reaching a wide population of potential subjects in different geographic areas. An enrollment target of approximately 250 subjects was arbitrarily selected for this pilot surveillance study, and the surveillance enrollment target was reached in approximately 1 week. Achieving the target enrollment of 248 subjects required that an approximately 10-fold larger number of parents enter the system, as only 16.71% (425/2543) completed the screening questionnaire. While the enrollment target was rapidly reached in this study, Internet-based recruitment is not always successful. Koo et al found a disappointing recruitment rate in a survey of teenagers and identified challenges, including concerns about the legitimacy of the website as a barrier to enrollment [12]. Similarly, Dobrow et al found that overall response rates to Internet-based surveys were low [13].

It is encouraging that more than half of the parents who entered the system eventually provided informed consent and enrolled in the study. This observation, coupled with the fact that the pool of subjects obtained with this Internet-based recruitment technology was generally representative of the US population in terms of race and ethnicity—white, black, and Hispanic or Latino subjects in this study 73.8% (183/248), 13.7% (34/248), and 9.3% (23/248), respectively, vs US population 77.9%, 13.1%, and 16.9%, respectively [14]—supports the use of such a strategy to enroll pediatric subjects in clinical studies. Along with the benefits, however, Internet-based surveillance strategies have challenges. Consideration should be given to the representativeness of the subject population with regard to socioeconomic factors. Education level of parents in this study, for example, tended to skew higher than has been reported for the US population. A greater percentage of parents in this study were high school graduates or higher compared to the US population, 98.4% (244/248) vs 85.7%, respectively, and a greater percentage held a bachelor’s degree or higher, 49.6% (123/248) vs 28.5%, respectively [14]. Also, network security, subject privacy, set-up costs, and operating costs need to be addressed when creating an Internet surveillance tool.

Ideally, studies designed to evaluate cough and cold therapies should recruit subjects and begin treatment within 24 hours of the appearance of symptoms [3,5]. However, only 26.4% (43/163) of parents who responded on the same day that symptoms appeared indicated they would be willing to bring their child to a clinical center to participate in a clinical study, and only 8.6% (14/163) reported that they would bring the child to a center the following morning. Among parents who responded either the same day or the following day that symptoms appeared, the proportion willing to bring the child to a clinical center increased to 54.0% (88/163). Thus, it appears that recruitment within 24 hours may be a significant barrier to overcome in the design of a study.

Another potential challenge that emerged from the survey results was low willingness to comply with clinical study requirements involving school and the need to record symptoms and administer study medication multiple times during the day. Only 12.3% (20/163) of parents indicated that they would permit their child to remain out of school to participate in a study. Although more parents, 29.4% (48/163), were willing to administer study drugs and collect symptom scores at school, these responses suggest that parental adherence to a study protocol that requires multiple doses during the day will be a major obstacle. In addition, there was a substantial decline in adherence to the protocol throughout this study. Fewer than half of all parents of enrolled children successfully completed the WCDS and DCSSS (43.5% (108/248) and 44.8% (111/248), respectively), and only 22.6% (56/248) completed all surveys.

### Conclusion

The results of this investigation suggest that Internet-based surveillance and recruitment can be useful tools to follow the natural history of colds in children and to enroll subjects in clinical studies of therapies for the treatment of cough and cold.

## Multimedia Appendix 1

Well child daily survey.

## Multimedia Appendix 2

Daily cold symptom severity survey.

## Multimedia Appendix 3

CHERRIES checklist.

## Abbreviations

DCSSS: Daily Cold Symptom Severity Survey
EOSS: End of Study Survey
WCDS: Well Child Daily Survey
