# Supplementary figures and images for: Common Cold Symptoms in Children: Results of an Internet-Based Surveillance Program
Source: J Med Internet Res. 2014 Jun 19;16(6):e144. doi: 10.2196/jmir.2868 (PMC4090373; doi:10.2196/jmir.2868)

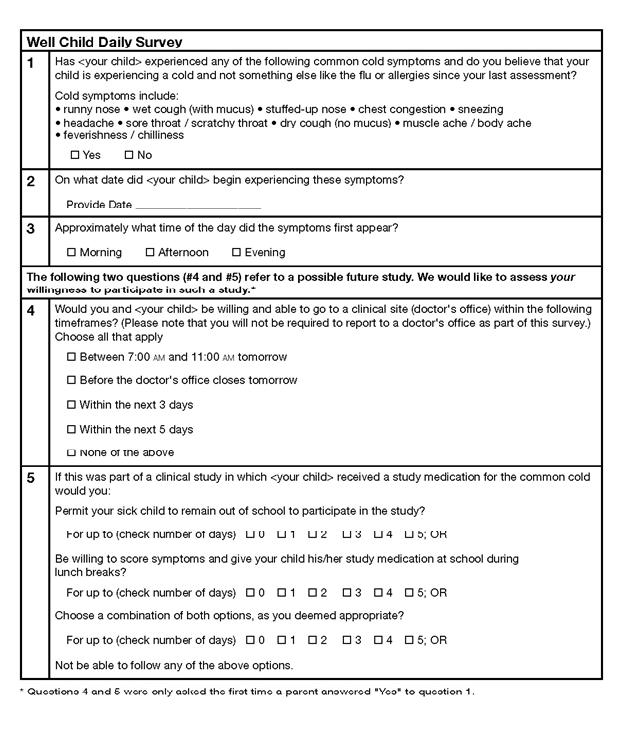

Supplement: Supplementary file 1 [file jmir_v16i6e144_app1.JPG]

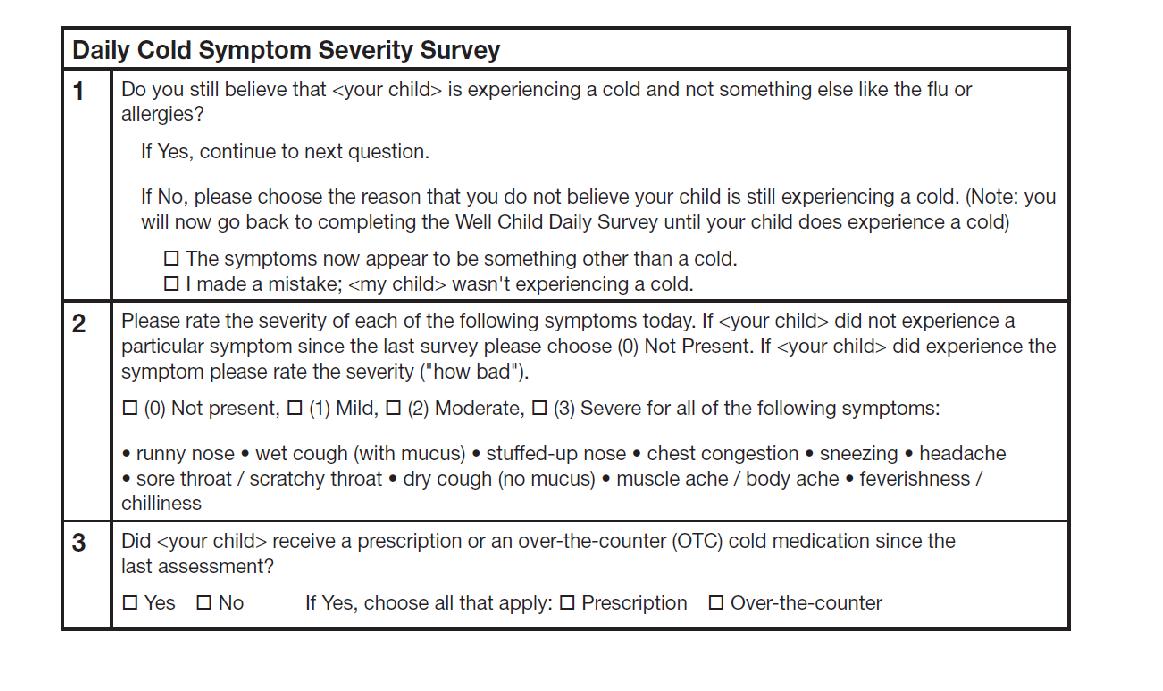

Supplement: Supplementary file 2 [file jmir_v16i6e144_app2.JPG]
