# Supplementary material for: Common Cold Symptoms in Children: Results of an Internet-Based Surveillance Program
Source: J Med Internet Res. 2014 Jun 19;16(6):e144. doi: 10.2196/jmir.2868 (PMC4090373; doi:10.2196/jmir.2868)
Supplement: Supplementary file 3 [file jmir_v16i6e144_app3.pdf]

**Design**		
	Describe survey design	Describe target population, sample frame. Is the sample a convenience sample? (In "open" surveys this is most likely.) This is a convenience sample.
**IRB (Institutional Review Board) approval and informed consent process**		
	IRB approval	Mention whether the study has been approved by an IRB. Study was approved by Copernicus Group, Independent Review Board
	Informed consent	Describe the informed consent process. Where were the participants told the length of time of the survey, which data were stored and where and for how long, who the investigator was, and the purpose of the study? Informed consent indicated the name of the investigator, the purpose of the study, length of study and number of participants, study procedures, potential risks and discomforts, benefits, costs to the participant, source of funding, reimbursement for participation, study-related injury, legal rights, confidentiality, voluntary participation/withdrawal, contacts, authorization to use and disclose medical information. The informed consent did not state how long the data would be kept.
	Data protection	If any personal information was collected or stored, describe what mechanisms were used to protect unauthorized access. Data protection was ensured through contractual agreement requirement for internet security. The contract for this project included an appendix to describe baseline third party security requirements. The addendum addresses the following: a) security levels to be applied by the Processor (the entity Processing the Pfizer information on behalf

		of Pfizer) to the types of information being processed; b) who is in charge of information security within the Processor; c) what organizational measures the Processor is required to implement; d) precautions to be taken by the Processor in relation to its staff and third parties; e) what physical security measures the Processor is required to implement; f) what computer security measures the Processor is required to implement; and, g) what actions are to be taken by the Processor in the event of a security breach.
**Development and pre-testing**		
	Development and testing	State how the survey was developed, including whether the usability and technical functionality of the electronic questionnaire had been tested before fielding the questionnaire.<br><br>The survey was developed by the CRO in conjunction with the Sponsor. A vendor was responsible for activities needed to initiate, maintain and close-out the electronic data capture system. Pfizer ensured the completion of User Acceptance Test (UAT) prior to publishing the survey online. UAT was completed by the service provider(INC) engaged by Pfizer.
**Recruitment process and description of the sample having access to the questionnaire**		
	Open survey versus closed survey	An “open survey” is a survey open for each visitor of a site, while a closed survey is only open to a sample which the investigator knows (password-protected survey).<br>This was a password-protected survey

	Contact mode	Indicate whether or not the initial contact with the potential participants was made on the Internet. (Investigators may also send out questionnaires by mail and allow for Web-based data entry.) Initial contact was made by letter to individuals who had previously agreed to receive information about clinical studies. Potential subjects were recruited through the resources of a patient recruitment provider (Acurian, Inc.) with an extensive patient database of opted-in patients. Acurian, Inc. was responsible for the following activities:<br>• Send 12,000 invitations to parents or legally authorized representative of children ages 6-11 years old to visit in their database<br>• The 12,000 invitations were distributed evenly across 4 regions of the U.S.: Austin, TX; Boston, MA; Portland, OR; Raleigh, NC<br>The letter directed the parent/LAR to an on-line website if they were interested in learning more about the study and also if they wanted to participate.
	Advertising the survey	How/where was the survey announced or advertised? Some examples are offline media (newspapers), or online (mailing lists – If yes, which ones?) or banner ads (Where were these banner ads posted and what did they look like?). It is important to know the wording of the announcement as it will heavily influence who chooses to participate. Ideally the survey announcement should be published as an appendix.<br>Letters were mailed by Acurian, Inc, a patient recruitment provider, mailed letters to individuals in their database informing them of the study and inviting them to visit a website if they wanted to learn more about the study or to participate. .
**Survey administration**		

	Web/E-mail	State the type of e-survey (eg, one posted on a Web site, or one sent out through e-mail). If it is an e-mail survey, were the responses entered manually into a database, or was there an automatic method for capturing responses? The survey was posted on a website. Parents entered data directly onto the website where it was electronically captured.
	Context	Describe the Web site (for mailing list/newsgroup) in which the survey was posted. What is the Web site about, who is visiting it, what are visitors normally looking for? Discuss to what degree the content of the Web site could pre-select the sample or influence the results. For example, a survey about vaccination on a anti-immunization Web site will have different results from a Web survey conducted on a government Web site The website was created specifically for the survey and was disabled at the completion of the project.<br>The website was a stand-alone website, for the use of survey participants. Parents who received a mailer informing them about the study were directed to a website where they could learn more about the study. They completed the pre-screening questionnaire if interested in participating. After satisfactory completion the prescreening questionnaire and informed consent/assent, the parent was asked to complete an on-line eligibility survey. After satisfactory completion the Eligibility survey, the parent was asked to complete an on-line registration/demographic survey.<br>Following successful completion of the registration survey, the parent was assigned a unique ID and password. The parent completed a well child daily survey each day to record the presence or absence of symptoms commonly associated with a cold. This survey was completed each day for 6 weeks or until the parent believed the

		child was experiencing a cold. When the parent believed that the child was experiencing a cold the parent was asked a few questions about the cold and then was directed to the Daily Cold Symptom Severity Rating Survey. The daily cold symptom ratings continued until all cold symptoms were completely resolved or for a maximum of 10 days, whichever came first. The parent completed an end of study survey after completing the Daily Cold Symptom Severity Rating Survey till the child was symptom-free or after the Day 10 survey, or after completing the well child survey for 6 weeks if the child never developed cold symptoms.
	Mandatory/voluntary	Was it a mandatory survey to be filled in by every visitor who wanted to enter the Web site, or was it a voluntary survey? The survey was voluntary
	Incentives	Were any incentives offered (eg, monetary, prizes, or non-monetary incentives such as an offer to provide the survey results)? A small monetary incentive (gift card) was offered to the parents completing the survey.
	Time/Date	In what timeframe were the data collected? There was a 6 week enrollment period to recruit subjects. Once enrolled, parents were asked to monitor their child for signs of a cold for up to 6 weeks.
	Randomization of items or questionnaires	To prevent biases items can be randomized or alternated. Everyone received the same questionnaire.
	Adaptive questioning	Use adaptive questioning (certain items, or only conditionally displayed based on responses to other items) to reduce number and complexity of the questions. Adaptive questioning was employed.
	Number of Items	What was the number of questionnaire items per page? The number of items is an important factor for the completion rate.

		Prescreen survey – 7 questions<br>Eligibility survey – 16 questions<br>Registration survey – 22 questions<br>Well Child survey – 5 questions<br>Cold Symptom survey – 12 questions<br>End of Study survey – 15 questions
	Number of screens (pages)	Over how many pages was the questionnaire distributed? The number of items is an important factor for the completion rate. There were 6 different survey forms but they were used at different times over the course of the 6 week period. The actual number of pages is unknown as screenshots were not taken.
	Completeness check	It is technically possible to do consistency or completeness checks before the questionnaire is submitted. Was this done, and if "yes", how (usually JAVAScript)? An alternative is to check for completeness after the questionnaire has been submitted (and highlight mandatory items). If this has been done, it should be reported. All items should provide a non-response option such as "not applicable" or "rather not say", and selection of one response option should be enforced. Documentation on whether or not a consistency/completeness check is unavailable.
	Review step	State whether respondents were able to review and change their answers (eg, through a Back button or a Review step which displays a summary of the responses and asks the respondents if they are correct). Documentation is not available regarding the review or ability to change answers.
**Response rates**		
	Unique site visitor	If you provide view rates or participation rates, you need to define how you determined a unique visitor. There are different techniques available, based on IP addresses or cookies or both. We didn't provide view rates. Participants were

		assigned unique identifier codes.
	View rate (Ratio of unique survey visitors/unique site visitors)	Requires counting unique visitors to the first page of the survey, divided by the number of unique site visitors (not page views!). It is not unusual to have view rates of less than 0.1 % if the survey is voluntary. Information on the view rate is not available.
	Participation rate (Ratio of unique visitors who agreed to participate/unique first survey page visitors)	Count the unique number of people who filled in the first survey page (or agreed to participate, for example by checking a checkbox), divided by visitors who visit the first page of the survey (or the informed consents page, if present). This can also be called "recruitment" rate. 425 completed the pre-screening questionnaire out of 2543 who entered the system. 425/2543 = 16.7%
	Completion rate (Ratio of users who finished the survey/users who agreed to participate)	The number of people submitting the last questionnaire page, divided by the number of people who agreed to participate (or submitted the first survey page). This is only relevant if there is a separate "informed consent" page or if the survey goes over several pages. This is a measure for attrition. Note that "completion" can involve leaving questionnaire items blank. This is not a measure for how completely questionnaires were filled in. (If you need a measure for this, use the word "completeness rate".) 109 completed the end of study survey out of 346 who completed the informed consent. 109/346 = 31.5%
**Preventing multiple entries from the same individual**		
	Cookies used	Indicate whether cookies were used to assign a unique user identifier to each client computer. If so, mention the page on which the cookie was set and read, and how long the cookie was valid. Were duplicate entries

		avoided by preventing users access to the survey twice; or were duplicate database entries having the same user ID eliminated before analysis? In the latter case, which entries were kept for analysis (eg, the first entry or the most recent)? Specific documentation is not available.
	IP check	Indicate whether the IP address of the client computer was used to identify potential duplicate entries from the same user. If so, mention the period of time for which no two entries from the same IP address were allowed (eg, 24 hours). Were duplicate entries avoided by preventing users with the same IP address access to the survey twice; or were duplicate database entries having the same IP address within a given period of time eliminated before analysis? If the latter, which entries were kept for analysis (eg, the first entry or the most recent)? Specific documentation is not available.
	Log file analysis	Indicate whether other techniques to analyze the log file for identification of multiple entries were used. If so, please describe. Specific documentation is not available.
	Registration	In “closed” (non-open) surveys, users need to login first and it is easier to prevent duplicate entries from the same user. Describe how this was done. For example, was the survey never displayed a second time once the user had filled it in, or was the username stored together with the survey results and later eliminated? If the latter, which entries were kept for analysis (eg, the first entry or the most recent)? Specific documentation is not available.
**Analysis**		
	Handling of incomplete questionnaires	Were only completed questionnaires analyzed? Were questionnaires which terminated early (where, for example, users did not go through all questionnaire pages) also analyzed? Data from completers and

		early terminators were analyzed. Completed and terminated questionnaires were analyzed.
	Questionnaires submitted with an atypical timestamp	Some investigators may measure the time people needed to fill in a questionnaire and exclude questionnaires that were submitted too soon. Specify the timeframe that was used as a cut-off point, and describe how this point was determined. Some time point cut-offs were used. Specific details are unavailable.
	Statistical correction	Indicate whether any methods such as weighting of items or propensity scores have been used to adjust for the non-representative sample; if so, please describe the methods. Only descriptive statistics were used; weighting of items or propensity scores were not used.
